# In vitro biochemical assessment of mixture effects of two endocrine disruptors on INS-1 cells

**DOI:** 10.1038/s41598-022-20655-0

**Published:** 2022-11-22

**Authors:** Lamidi W. B. Olaniyan, Anthony I. Okoh

**Affiliations:** 1 Biochemistry Department, Faculty of Basic Medical Sciences, Ladoke Akintola University of Technology, Ogbomoso, Nigeria; 2 SAMRC-Microbial Water Quality Monitoring Centre, University of Fort Hare, Alice, 5700 South Africa

**Keywords:** Biocatalysis, Enzyme mechanisms, Biochemistry, Biological techniques, Biotechnology, Molecular biology, Risk factors

## Abstract

4-tert-Octylphenol (4-tOP) is a component of non-ionic surfactants alkylphenol polyethoxylates while triclosan (TCS) is an antibacterial present in personal care products. Both compounds can co-exist in environmental matrices such as soil and water. The mixture effects of these micropollutants in vitro remains unknown. INS-1 cells were exposed to 20 µM or 30 µM 4-tOP and 8 µM or 12.5 µM TCS as well as equimolar mixture of the chemicals (Mix) in total concentration of 12.5 µM or 25 µM for 48 h. Mitochondrial related parameters were investigated using high content analytical techniques. The cytotoxicity of the chemicals (IC_50_) varied according to TCS > Mix > 4-tOP. Increased glucose uptake and loss of mitochondrial membrane potential were recorded in TCS and Mix treated cells. Fold values of glucose-galactose assay varied according to dinitrophenol > TCS > 4-tOP > Mix in decreasing order of mitochondrial toxicity. The loss of the intracellular Ca^2+^ influx by all the test substances and Mix was not substantial whereas glibenclamide and diazoxide increased the intracellular Ca^2+^ influx when compared with the Blank. The recorded increase in Ca^2+^ influx by diazoxide which contrasted with its primary role of inhibiting insulin secretion need be re-investigated. It is concluded that the toxic effects of TCS and Mix but not 4-tOP on INS-1 cells was mitochondria-mediated.

## Introduction

4-tOP is a component of non-ionic surfactants alkyl phenol polyethoxylates while TCS is an antibacterial present in personal care products. The two compounds can co-exist in environmental matrices and their high levels have been recorded thereby affording humans and wildlife sufficient exposure for toxicity. The compounds are environmental toxicants following their demonstrated endocrine disruption^1,2^. Data are accumulating linking environmental factors via endocrine disrupting molecules to the increase in metabolic syndrome^3^. Cells such as β-cells which express oestrogen receptors are affected by endocrine disruptors such as 4-tOP and TCS^4^. Reports of Ajao et al.^5^ and Weatherly et al.^6^ suggested that TCS targets mitochondria in the β-cells but that of 4-tOP with respect to mitochondrial toxicity has not been precisely defined. Following the two compounds ability to coexist in environmental matrix, the resulting toxicity in vitro or in vivo deserves elucidation. INS-1, the rat insulinoma cell line has been widely applied as an in vitro model for β-cell metabolism. We have used the model to present biochemical evidence of toxicity in INS-1 cells by the two household endocrine disruptors individually and in mixture.

## Materials and methods

Rat insulinoma cell line INS-1 cells were obtained from the Department of Biochemistry and Microbiology Nelson Mandela Metropolitan University, South Africa. RPMI-1640, 4-tOP (97%; CAS 140-66-9), TCS (99%; CAS 3380-34-5), glucose oxidase reagent (RANDOX Kit) and other chemical reagents were available commercially and were of analytical grade. 1 mM CellROX orange oxidative stress reagent stock solution was prepared in dimethyl sulphoxide (DMSO). For CellROX staining solution, to 10 mL of phosphate—buffered saline (PBS), were added 20 µL CellROX stock and 2 µL Hoechst 33342 solution (10 mg/mL in DMSO). For thiol tracker, 20 mM ThiolTracker Violet stock solution was prepared in DMSO. To 10 mL of PBS were added 1 µL Thioltracker Violet stock and 20 drops NucRed as thiol tracker staining solution. Propidium iodide (PI) was prepared in binding buffer to a final concentration of 2 µg/mL; 5 mL binding buffer contained 50 µL Annexin V-FITC (fluorescein isothiocyanate) reagent (Milteny Biotec Annexin V-FITC Kit Cat no. 130-092-052). Stock Tetramethylrhodamine ethylester (TMRE) solution was 5 mg/mL in DMSO. Working solution of TMRE was prepared by diluting 1 µL stock to 200 µL in RPMI medium.

### Maintenance of INS-1 cell line

INS-1 cells were routinely grown at 5% CO_2_, 95% air at 37 °C in RPMI-1640 containing 11.1 mM glucose and supplemented with 10% foetal calf serum (FCS), 1 mM pyruvate, 10 mM 4-(2-hydroxyethyl)-1-piperazine ethane sulphonic acid (HEPES), 50 µM 2-mercaptoethanol, 100 U penicillin/mL and 100 µg streptomycin/mL. Sub-confluent cultures were trypsinized to dislodge the cells and sub-cultured at a ratio of 1:3 or 1:0.75 in other experiments. INS-1 cells were seeded into a 96-well plate at densities of 10 000, 15 000 or 30,000 cells/well. They were allowed to attach at 37 °C overnight.

### Treatment of INS-1 cells

The cells were incubated for 48 h with test compounds containing 20 µM or 30 µM 4-tOP and 8 µM or 12.5 µM TCS. Combination treatments (4-tOP + TCS) were 12.5 µM or 25 µM at 1:1 ratio. Melphalan (40 µM) and Etoposide (40 µM) were used as positive controls in some experiments.

### Cell viability assay

Cell viability was assessed by a principle of enzymatic reduction of 3-[4, 5-dimethylthiazole-2-yl]-2, 5-diphenyltetrazolium bromide (MTT) to formazan. The treated cells respectively were loaded with 20 µL 0.5 mg/mL MTT and incubated at 37 °C for 3 h. The MTT was removed and 200 µL DMSO/well added to solubilize the formazan crystals. The absorbance of the purple-coloured formazan was measured at 540 nm on microplate reader. IC_50_, (the median inhibitory concentration) was calculated with GraphPad Prism software. Data were expressed as a percentage of control.

### Glucose-galactose cytotoxicity assay

Sub-culturing was in ratio 4:3. Cell density was 15,000 cells/well and treated with 200 µL of the test solution that was solubilized in glucose-free RPMI medium supplemented with 5 mM glucose or 5 mM galactose. The cells were incubated further for 48 h. The spent medium was replaced with 100 µL glucose in RPMI medium. 2,4-dinitrophenol served as positive control. The cells were finally loaded with MTT as in section “Cell viability assay” but incubation took 120 min.

### Glucose uptake assay

Glucose uptake was determined in INS-1 cells by monitoring the levels of glucose remaining in the culture medium after a selected treatment period. The cell density was 30 000 per well; one row was without cells to serve as maximum glucose content. Spent medium was removed and the cells were treated with 100 µL of the test compounds at concentrations indicated in section “Treatment of INS-1 cells”. The cells were incubated for a further 48 h and 5µL spent culture medium was thereafter transferred to a new plate of 96 wells and 200 µL glucose oxidase reagent added. Incubation was at 37 °C for 20 min and absorbance was read at 520 nm. Glucose uptake was obtained as the difference in glucose concentration between wells without cells and those with cells and respective treatments.

### Oxidative stress determination

The spent medium in section “Treatment of INS-1 cells” was removed and 50 µL CellROX staining solution was added to each well according to the manufacturer’s instructions and incubated at 37 °C for 30 min. Images from nine sites per well were acquired using filter sets appropriate for DAPI (4′, 6-diamidino-2-phenylindole) and TRITC (tetramethylrhodamine-isothiocyanate) which were the fluorophors. Image acquisition was done on ImageXpress Micro XLS and analyzed using the Multi-Wavelength Scoring software (Molecular Devices). In another experiment 50 µL ThiolTracker Violet staining solution was put in each well and incubated at 37 °C for 20 min. Images from nine sites per well were acquired using filter sets appropriate for DAPI and CY5 (amine reactive fluorescent dye). Image acquisition and data analysis were as done for the CellROX orange reagent. Excitation/Emission wavelength was at 545/565 nm.

### Assessment of apoptosis in the cells

#### Annexin V- FITC, PI and Hoechst staining assay

The treated cells were sub-cultured at 4:3. Annexin V-FITC staining assay was performed as recommended by the manufacturer. Melphalan (Mel) and Etoposide (Etop) served as the positive controls. Incubation lasted 48 h. The spent medium was removed and 50 µL PI staining solution was in each well and incubated for 15 min at 37 °C followed by image acquisition and data analysis as in previous experiments. Images from nine sites per well were acquired using filter sets appropriate for DAPI, FITC and Texas red dyes.

#### Caspase-3 activation assay

Additional 100 µL test compounds were solubilized in the medium and incubated for 48 h. The spent medium was replaced with 100 µL of fix solution (4% formaldehyde in phosphate-buffered saline (PBS) and was fixed overnight; the cells were permeabilized with ice cold methanol, then washed with PBS and blocked using 1% BSA followed by incubation with the primary antibody at 1:200 dilution in blocking buffer for 2 h. The excess antibody was removed by washing with blocking buffer. Incubation was thereafter carried out with secondary antibody (FITC labelled) for 30 min and later washed with blocking buffer to remove excess antibody. Images from nine sites per well were acquired using filter sets appropriate for DAPI and FITC. Image analysis was done as in previous experiments.

### Determination of mitochondrial mass

Staining solution consisted of 10 mL of PBS to which was added 1 µL MitoTracker Green (MTG) stock (1 mM in DMSO) and 2 µL Hoechst solution (10 mg/mL in DMSO). Spent medium was removed and 50 µL staining solution added to each well (96 wells) followed by incubation at 37 °C for 20 min.

### Analysis of mitochondrial membrane potential

TMRE is a cell permeable cationic dye that accumulates in the mitochondrial matrix according to mitochondrial membrane potential (ΔψM). Staining solution was prepared by adding 5 mL PBS, 50 µL TMRE working solution and 2 µL Hoechst 33342 solution to 5 mL of RPMI. The spent medium was removed and 50 µL staining solution was put in each well and incubated for 30 min at 37 °C. Image acquisition and data analysis were done as indicated.

### Analysis of glucose stimulated calcium influx

Fluo-4 dye is specific for intracellular Ca^2+^ concentration. On binding calcium, the green fluorescence intensity increases by more than 100 folds (excitation/emission 494/506 nm). Incubation was for 24 h. Staining solution was prepared by adding 20 µL Flou-4 stock solution (1 mM in DMSO) and 2 µL Hoechst solution to 10 mL of PBS. After staining, incubation was done for 30 min at 37 °C. The stain solution was removed and replaced with 50 µL PBS. Incubation was further carried out at room temperature for 20 min. Difference in fluorescence intensity between wells containing glucose and those without glucose represents calcium influx in response to glucose. Just prior to image acquisition, 20 mM glucose was added to four of the eight replicate wells and incubated at room temperature for 10 min. Image acquisition and data analyses were done as usual. Filter sets appropriate for DAPI and FITC were also used. Diazoxide and Glibenclamide were used as the positive controls.

## Statistical analysis

Results were expressed as means ± SD from at least three replicate measurements. The difference between means was analyzed with ANOVA, α value = 0.05. Post-hoc analysis was done using Tukey test. IC_50_ or median inhibitory concentration was defined as the concentration of the compound which caused 50% response, calculated using GraphPad Prism software 9, 2022.

## Results

### Cytotoxicity

Preliminary investigation showed that the test compounds had suitable solubility properties for cell-based assays. Both compounds were completely soluble in DMSO up to 50 mM.

**Figure 1:**
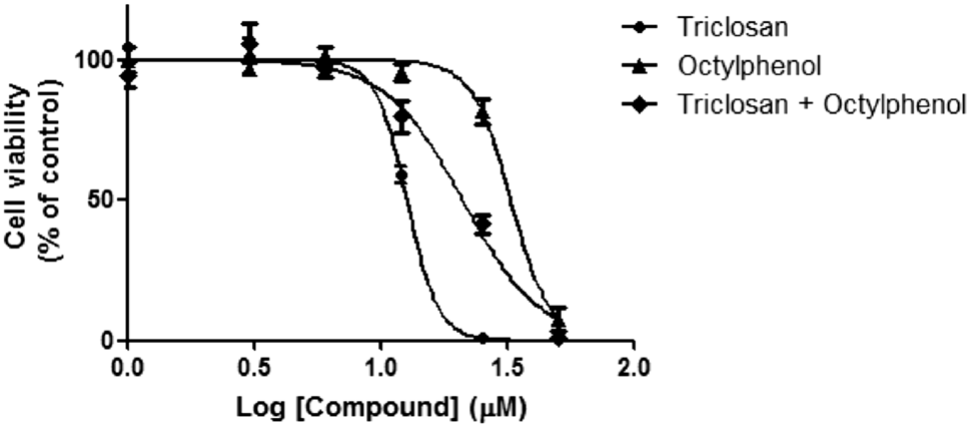
Concentration dependent cytotoxicity of 4-tert-octylphenol, triclosan and equimolar ratio mixture of both compounds in INS-1 cells treated for 48 h.

Figure 1 and Table 1 depict the cell viability in the presence of single or combined concentrations of the test compounds after 48 h. IC_50_ value was lowest in triclosan treated group. Each compound induced loss of cell viability at concentration above 50 μM.

**Table 1:** IC_50_ values for cytotoxicity against INS-1 cells.

Test compound	IC_50_ (μM)	R^2^
4-tert-Octylphenol	32.51	0.98
Triclosan	12.65	0.99
4-tert-Octylphenol: triclosan (1:1)	21.01	0.97

**Figure 2:**
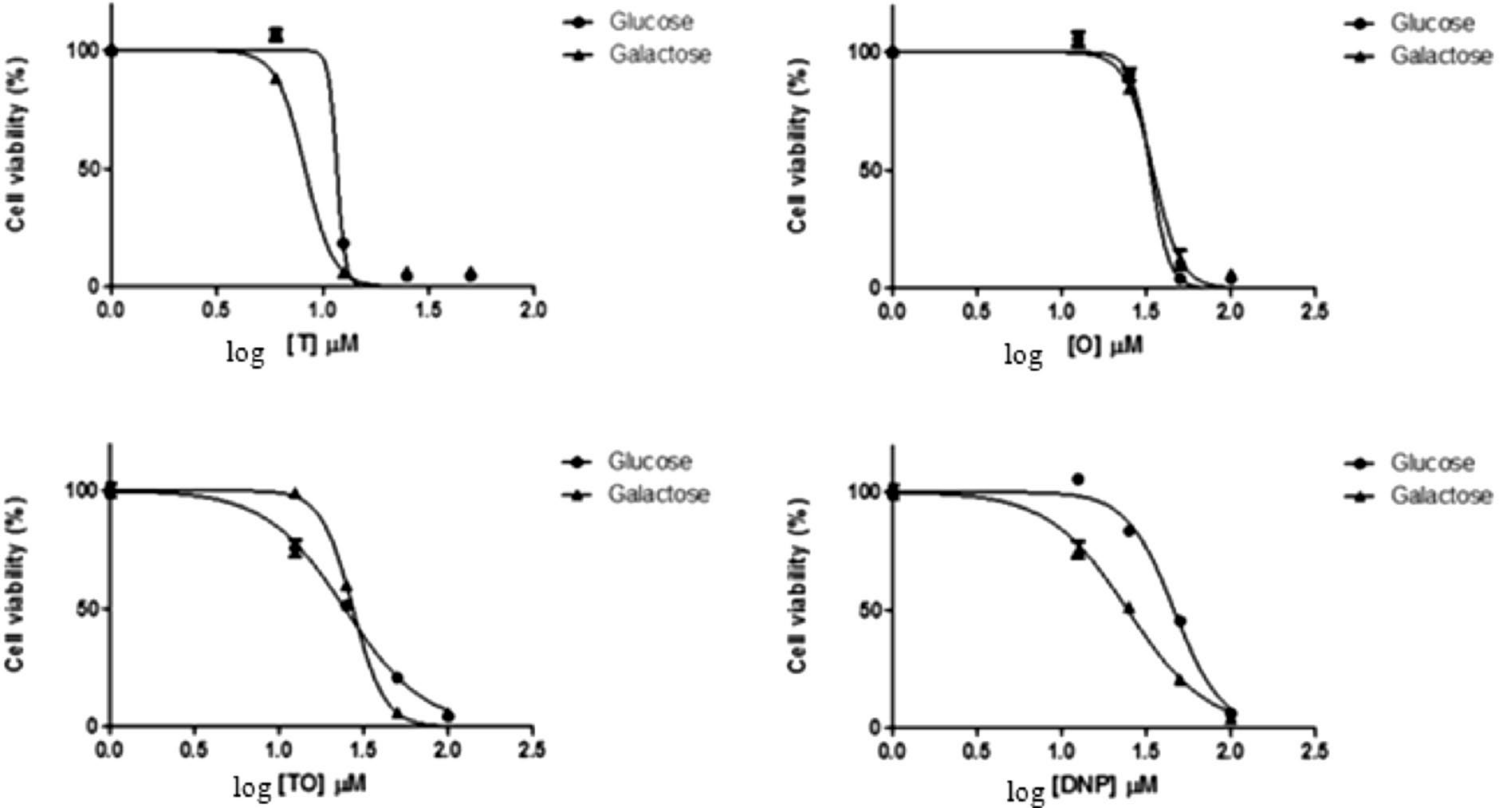
Differential cytotoxicity of INS-1 cells cultured in glucose or galactose medium. INS-1 cells were treated with varying concentrations of Triclosan, 4-tert-Octylphenol, 1:1 molar Triclosan: 4-tert-Octylphenol and 2, 4-Dinitrophenol (DNP).

Differential cytotoxicity of the three treatments in glucose versus galactose medium was reported in Fig. 2, which yielded the IC_50_ values listed in Table 2. Both TCS and DNP exhibited higher toxicity in the galactose medium than in the glucose medium while 4-tOP and the combination exposure (Mix) presented a reverse case though of little difference. All the IC_50_ values showed very high positive correlation (R^2^ > 0.9) (Table 2).

**Table 2:** IC_50_ values of treated INS-1 cells in glucose- or galactose-containing culture medium.

Compound	IC_50_ (μM) Glucose	IC_50_ (μM) Galactose	Glu- IC_50_/Gal- IC_50_)
Triclosan	11.67	8.23	1.42
4-tert-Octylphenol	33.41	34.54	0.97
Triclosan: 4-tert-Octylphenol (1:1)	24.48	27.34	0.90
2,4-Dinitrophenol (DNP)	46.03	24.48	1.88

**Figure 3:**
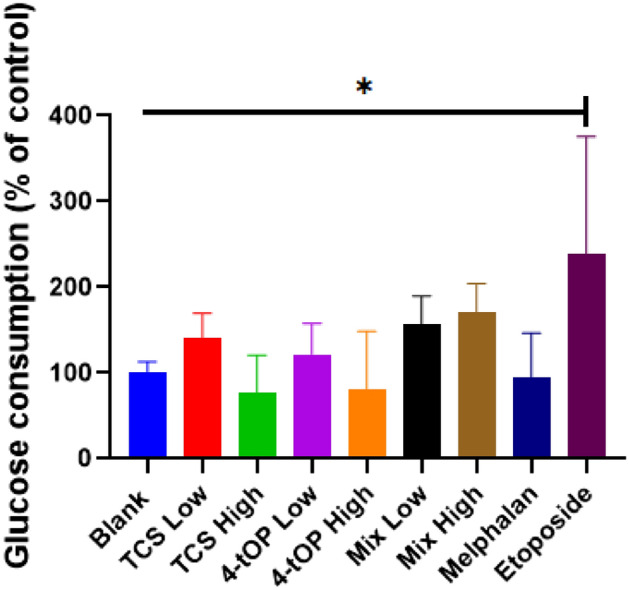
Effects of the test substances on cellular glucose uptake. p value* = 0.021, n = 5. TCS High/Low, Triclosan High/Low concentration; 4-tOP High/Low, 4-tert-Octylphenol high/low concentration; Mix High/Low, TCS + 4-tOP high/low concentration.

Figure 3 shows the trend of glucose consumption by INS-1 cells. Glucose consumption appeared to be concentration dependent; increased uptake was induced by low concentration of the test substances and vice versa, relative to the Blank. The mixtures (Mix) presented a reverse scenario in which those cells exposed to the high concentration of Mix displayed high glucose uptake and low Mix evoked reduced glucose uptake. These conditions were however not statistically significant when compared with the Blank (Fig. 3). Only cells exposed to Etop showed significant increase in glucose uptake relative to the Blank.

**Figure 4:**
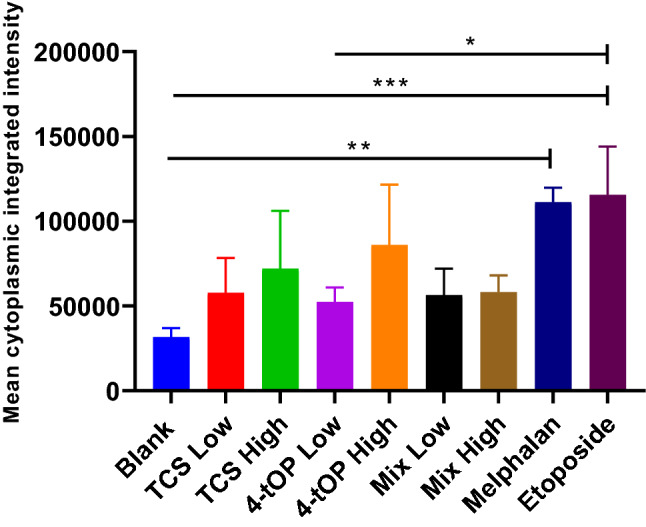
Estimates of the activity of the reactive oxygen species. p values * = 0.04, ** = 0.0064, *** = 0.0038, n = 3.

CellRox reagent kit estimated the activity of reactive oxygen species (ROS) (Fig. 4). There was a general increase in the activity of ROS in the cells exposed to the test compounds or the positive controls relative to the Blank samples. The activity of the ROS also appears to be concentration dependent among cells exposed to TCS, 4-tOP and their mixtures but the differences were not substantial when compared with the Blank. Cells exposed to low 4-tOP showed significantly reduced ROS activity when compared with Etop unlike TCS and the mixtures. The two positive controls induced substantially higher ROS activity than the Blank (Fig. 4).

**Figure 5:**
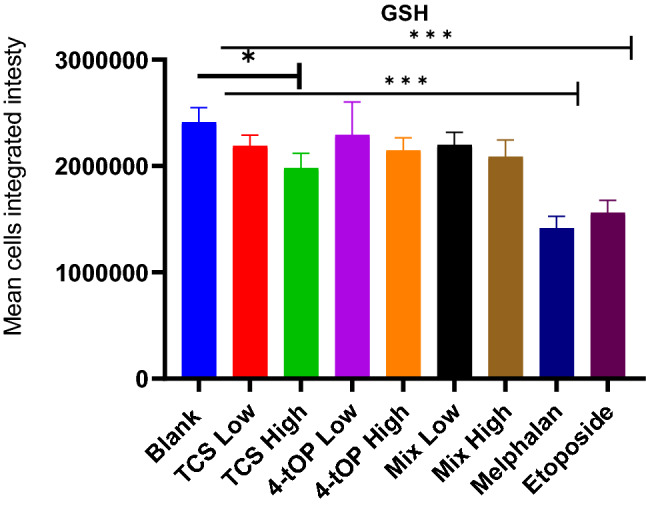
Glutathione concentration. p values * = 0.0157, *** = 0.0001 n = 4.

The cellular levels of reduced glutathione (GSH) were estimated using ThioltrackerViolet dye in evaluating oxidative stress (Fig. 5). As observed with ROS, GSH metabolism in the cells treated with the test compounds or their mixtures (Mix) appeared (p > 0.05) to be concentration dependent when compared with the Blank (Fig. 5). There was a general reduction of GSH in the exposed cells. Only high TCS concentration (p = 0.0157) as well as the two positive controls, Mel (p = 0.0001) and Etop (p = 0.0001) showed significant reduction of GSH when compared with the Blank. However, all the test substances or their Mix showed significant increase in GSH concentration relative to each of the two controls.

**Figure 6:**
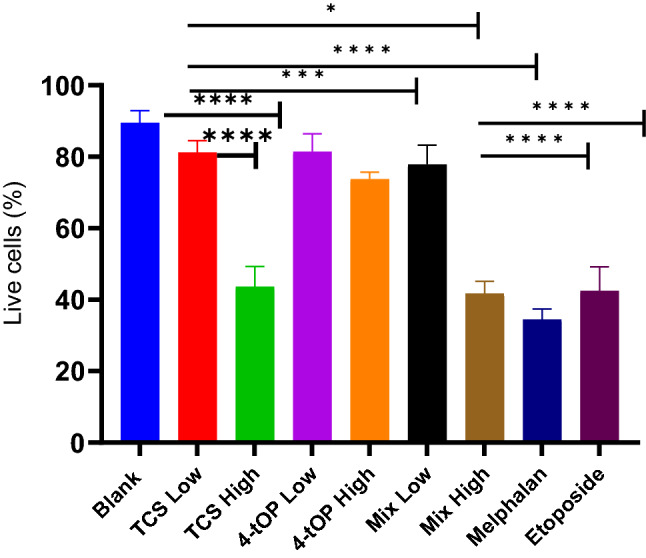
Proportion of live cells after treatments. p values * = 0.023, *** = 0.0009, * = 0.0001, n = 4.

Mechanism of cell death was studied using Annexin V-FITC, PI and Hoechst staining techniques (Fig. 6). The high proportions of live cells remaining after administration of TCS (81.2%) and 4-tOP (81.4%) at their low concentrations when compared with the Blank were not statistically significant (p = 0.22 and 0.24 respectively) (Fig. 6). Significantly reduced live cells were recorded following administrations of high concentration of TCS (43.7%, p = 0.0001) and of 4-tOP (73.8%, p = 0.0009), low (77.9%, p = 0.022) or high (41.7%, p = 0.0001) concentration of Mix as well as Mel (34.5%, p =  < 0.0001) and Etop (42.5%, p =  < 0.0001) when compared with the Blank. The reduction of live cells from 81.2% to 43.7% by TCS was strongly significant (p =  < 0.0001), such substantial reduction was not recorded for cells exposed to 4-tOP. For the Mix, the reduction of the live cells following increase in concentration was substantially significant (p =  < 0.0001). Cell reduction by the low Mix but not by the high Mix, was significant when compared with Mel or Etop (p =  < 0.0001). Although Mel reduced more live cells than Etop at equal concentrations, the difference was not significant (p = 0.26) (Fig. 6).

**Figure 7:**
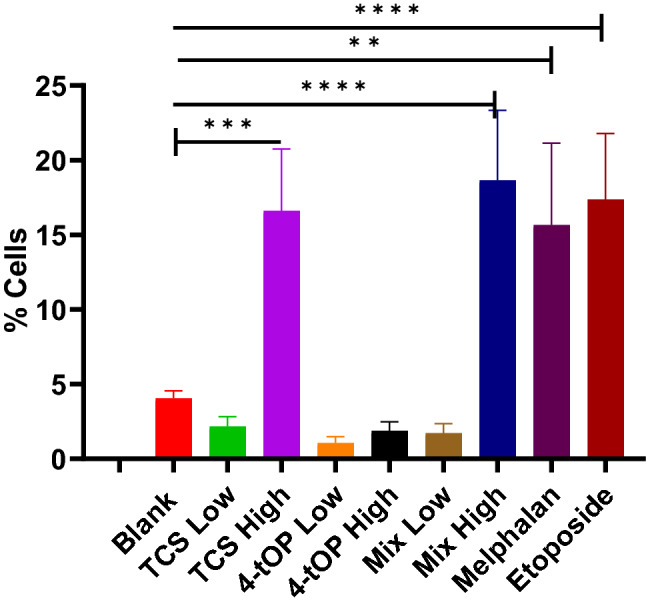
Proportion of cell death by apoptosis following treatments. p values *** = 0.0002, ** = 0.0006, **** = 0.0001, n = 4.

Significantly higher proportion of cells exposed to TCS (16.6%) and Mix (18.6%) at their high concentrations as well as Mel (15.7%) and Etop (17.4%) died by apoptosis than the Blank (Fig. 7). 4-tOP failed to significantly induce apoptosis in the cells when compared with the Blank.

**Figure 8:**
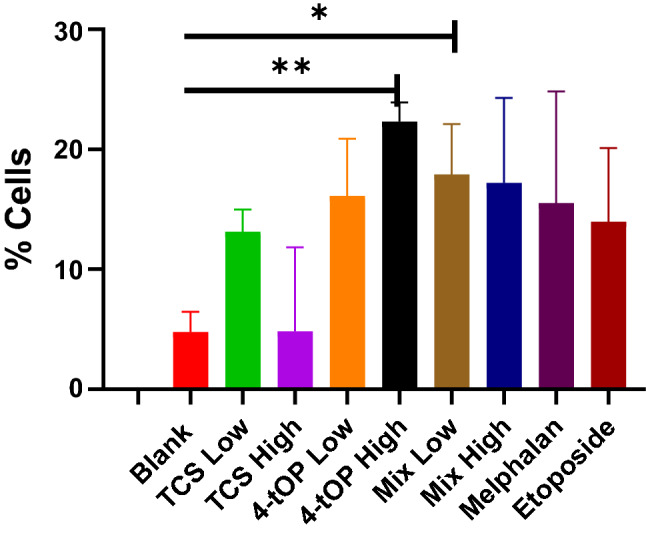
Proportion of cell death by necrosis after treatments. p values * = 0.048, ** = 0.0032, n = 4.

Only cells exposed to the high concentration of 4-tOP and to the low concentration of Mix significantly underwent necrosis when compared with the Blank (Fig. 8).

**Figure 9:**
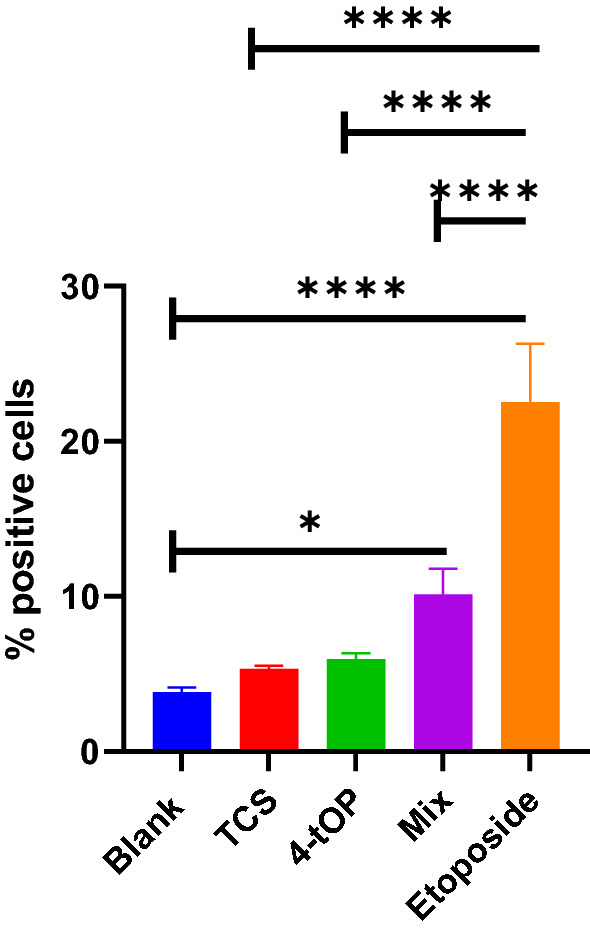
Caspase 3 activation. p values * = 0.012, **** = 0.0001, n = 3.

Figure 9 illustrates caspase 3 activation. There were significant increases in caspase 3 activation by Mix and Etop when compared with the Blank samples. But marked reductions of caspase 3 activation by the three test substances, TCS, 4-tOP and Mix (p = 0.0001) were recorded when compared with Etop.

**Figure 10:**
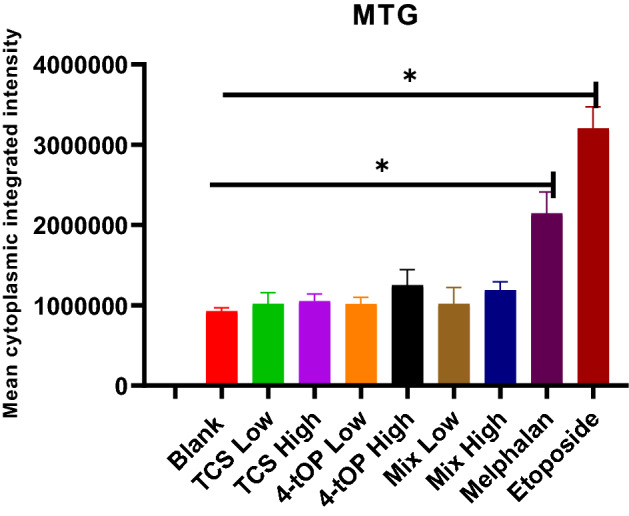
MTG-Mitotracker green staining for the determination of the effect of the test substances on mitochondrial mass. p values * = 0.0001, n = 4.

MTG staining investigated changes in the mitochondrial mass (Fig. 10). Only Mel and Etop significantly increased mitochondrial mass relative to the Blank. The increased mitochondrial mass by Etop was significantly different (p = 0.0001) from Mel. The differences between the test substances and the positive controls (Mel and Etop) were substantial (p =  < 0.0001).

**Figure 11:**
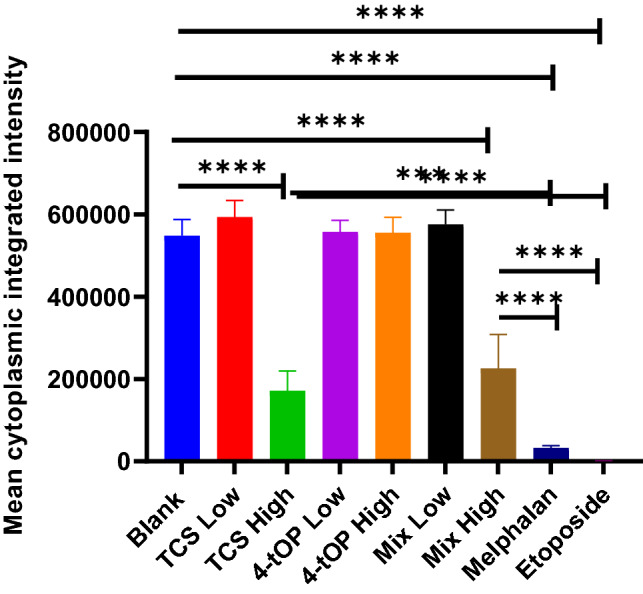
Changes in mitochondrial membrane potential in the cells exposed to the test substances. p values **** = 0.0001, Mel *** = 0.0009, Etop **** = 0.0001, n = 4.

Significant loss of mitochondrial membrane potential ΔΨM (MMP) was recorded at the high concentrations of both TCS and Mix as well as Mel and Etop compared to the Blank (Fig. 11).

**Figure 12:**
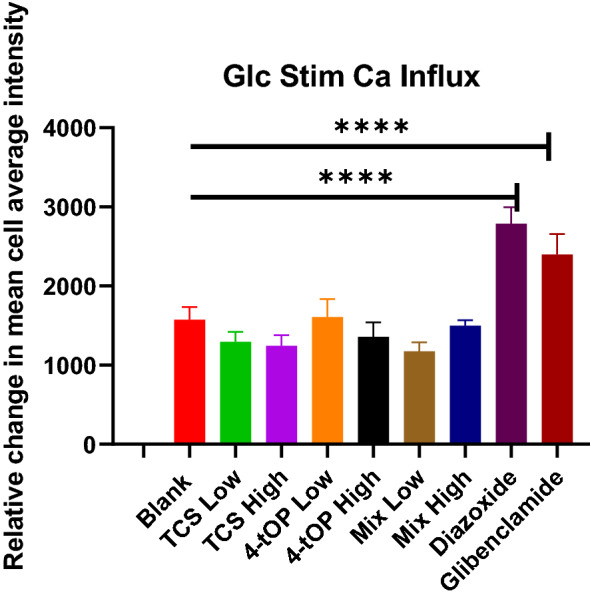
Effect of the test substances on glucose stimulated intracellular calcium influx. Diazoxide and Glibenclamide as positive controls. p values **** = 0.0001, n = 4.

The loss of MMP by Mel and Etop was very substantial when compared with TCS or Mix (Fig. 11). In the analysis of glucose stimulated intracellular calcium influx (Fig. 12). Diazoxide and Glibenclamide significantly increased intracellular calcium flux when compared with the Blank in direct contrast to the test substances which reduced the flux.

## Discussion

High content analysis or high content screening (HCA/HCS) is an automated imaging-based methodology used to assess cellular-based toxicity^7,8^. The cytotoxicity test aside testing for cell viability, also determined the affordability of studying the selected compounds in an in vitro biological system comprising INS-1 cells in DMSO as a medium^9^. Reduction of MTT occurs by mitochondrial dehydrogenase in active cells, the level of activity is therefore a measure of the viability of the cells. The result showed that TCS was more reactive to INS-1 cells than HepG2 human liver cells (IC_50_ 24.5 μM)^10^. The toxicity of the combination was lower compared to that of TCS but increased toxicity compared with that of 4-tOP, meaning that the mixture effect was probably antagonistic^4,11–13^. It may be that the constituent compounds attacked different molecular targets^14^.

Oxidation of galactose to pyruvate via glycolysis yields no net ATP forcing cells to rely on mitochondrial oxidative phosphorylation (the Crabtree effect) to produce sufficient ATP for survival^15,16^. Mitochondrial toxins display greater toxicity when cultured in galactose medium than in glucose medium and hence a ratio (fold value) greater than 1 is indicative of potential mitochondrial toxicity^17,18^ meaning that TCS as well as DNP is a mitochondrial toxin supporting previous reports that both compounds are mitochondrial uncouplers^6,19^. The fold value for 4-tOP and TO was less than 1 implying negative response to mitochondrial toxicity. The observed increase in glucose consumption by 4-tOP and Mix was therefore an adaptive or compensatory mechanism rather than a mitochondrial toxicity.

Glucose is the major fuel source for cells; the rate of glucose consumed by cells may serve as a reflection of their metabolic activity^20^. Etop, a topoisomerase II inhibitor is a medication for the management of various cancers such as testicular, prostate, bladder, stomach, and lung cancer^21^. It was the only test compound that sufficiently enhanced glucose uptake by the cells, possibly by up-regulation of glucose transporter or hexokinase activation. Glucose uptake by INS-1 cells, is thought to be predicated on the expression of GLUT 2, phosphorylation of insulin receptor (IR), insulin receptor substrate-1 (IRS1), phosphatidylinositol-3-kinase (PI3K) and AKT^22^. The increased uptake could be primarily a compensatory or adaptive response to produce sufficient ATP from glycolysis so as to sustain cell viability^17^. In addition, glucose metabolism via the pentose phosphate pathway links the antioxidant ability of the cells to glucose consumption^23^; consequently, enhanced rates of glucose metabolism might also reflect a survival strategy against oxidative insult. Increase in cellular glucose consumption may suggest mitochondrial involvement in the cell death mechanisms^17^ because mitochondrial toxins elevate cellular glucose uptake^24^. TCS, 4-tOP, their mixtures (Mix) and Mel respectively at their various concentrations did not produce a definitive effect on glucose uptake by the cells. Their respective reduction in glucose uptake failed statistical significance when compared with the Blank. Decreased glucose uptake as seen in skeletal muscles of type 2 diabetes following insulin resistance is thought to be caused by a defect in insulin signalling or abnormal deposition of transporter in the membrane compartments^25^.

CellROX oxidative stress reagents are fluorogenic probes for detecting reactive oxygen species (ROS) in live cells. ROS which include superoxide anion (O^2·−^) and H_2_O_2_ are generated by aerobic cells during electron transfer in the mitochondria. The role of ROS in β-cell function is complex as ROS also regulate insulin release^26^. GSH is a cellular antioxidant molecule which participates in catalytic cycles of antioxidant enzymes such as glutathione peroxidase and glutathione reductase. The substantial reduction of GSH concentration by high TCS was probably a response to the oxidative challenge. The increased activity of ROS with a corresponding reduction in GSH concentration recorded in the cells exposed to low 4-tOP, Mel and Etop might be that the cells were under oxidative stress^27–29^. Following extreme sensitivity of beta cells to oxidative attack^30^, it is customary to supplement INS-1 cell culture medium with antioxidants such as GSH. This was added to the RPMI medium at 3.2 µM. It might thus be expected that such condition could lead to an under-estimation of pro-oxidant effects of the test compounds when assessed in vitro. The consequences of redox imbalance are membrane lipid peroxidation, DNA damage, protein oxidation and interference of signal transduction which contribute significantly to beta-cell dysfunction and death. Oxidative stress leads to apoptosis by activating the intrinsic apoptosis pathway^31^. Detection of cell surface phosphatidylserine (PS) by Annexin V, a 35 kDa Ca^2+^—dependent phospholipid binding protein with very high affinity (K_D_ = 7 nM) is the methodology of choice for detecting apoptosis^32^. Annexin V binding assay detects early phases of apoptosis on cellular membrane level before the loss of cell membrane integrity^33^. Loss of phospholipid asymmetry is an early phenomenon in apoptosis^34^. The test discriminates intact live cells (FITC−/PI−), apoptotic cells (FITC+/PI−) and necrotic cells (FITC+/PI+). Of the three classes of the test substances (TCS, 4-tOP, Mix), the proportion of dead cells were comparable with high TCS concentration and high Mix lending credence to the result of the viability test. The results from PS asymmetry seemed to suggest that TCS and Mix induced cell death by apoptosis while 4-tOP was by necrosis. However, 4-tOP has been reported to induce apoptosis in the neuronal progenitor cells in mouse brain^35^, in rat Sertoli cells^36^, in the testicular germ cells of prepubertal rats^37^, and in human embryo stem cells^38^ showing that the compound operates different toxicity mechanisms in different cells as presumed for TCS^39,40^.

Imbalance in the expression of pro- and anti-apoptotic proteins following induction by stimuli is the underlying mechanism of cells undergoing apoptosis. In multicellular organisms, cellular death occurs by apoptosis and necrosis. Apoptosis is triggered by activation of death receptors (tumour necrosis factor receptor superfamily) located on the cell membrane surface (extrinsic pathway) or by disrupting intracellular homeostasis namely, DNA damage, cellular stress (intrinsic pathway)^41,42^. Cell necrosis is cell death associated with injury following an encounter with noxious stimuli such as environmental stressors like chemicals and extremes of environmental conditions such as temperature, radiations, and hypoxia or infectious agents like bacteria and viruses. During the initial stages of apoptosis, the cell membrane remains intact, while at the very moment that necrosis occurs the cell membrane loses its integrity and becomes leaky^43^. The significant increase in caspase 3 activation by Etop appear to confirm it as a trigger of caspase-mediated apoptotic pathways^44^. Similarly, the induction of apoptosis by Mix was caspase-mediated while apoptosis by TCS was caspase-independent exemplified by the apoptosis-inducing factor protein activity^45^. Caspase-3 is an effector enzyme in apoptosis which is activated by caspase-9 holoenzyme (caspase-9 + apoptosome)^46^. Caspase-9 is apoptosis initiator. Both caspases are regulated by Inhibitor of Apoptosis protein^47^. The activation is on the intrinsic pathway in which mitochondria are thought to play an important role^42^.

MTG is a green-fluorescent (excitation/emission 490/520 nm) mitochondria-selective stain believed to localize to mitochondria regardless of mitochondrial membrane potential^48^. The dye binds covalently with mitochondrial proteins through reaction with free sulphydryl groups of cysteine residues^49^. The increased staining by this dye therefore suggests increased mitochondrial staining. The increased mitochondrial mass as recorded with the positive controls suggests an increase in mitochondrial protein^50,51^ which may constitute a cell endogenous response to compensate for the mitochondria-related ‘deficits’^52^. It appears all the test substances at the concentrations applied, had no substantial effect on the mitochondrial mass.

The significant loss of MMP by TCS, Mix, Mel and Etop-exposed cells suggests the involvement of mitochondria in the apoptosis^53,54^. Failure of 4-tOP to reduce MMP affirms the substantial difference in the mechanism of toxicity between the two compounds (TCS and 4-tOP) in INS-1 cells. Loss of MMP leads to an opening of the mitochondrial permeability transition pore and subsequent leakage of intermembrane proteins, including cytochrome C that facilitates the induction of apoptosis through apoptosome formation^55^.

To evaluate the functionality of the β-cells in the presence of the compounds, glucose- stimulated insulin secretion was selected as a molecular signature to assess intact β-cells specific function. To this end, an in vitro system was established in which changes in the cellular calcium levels with or without glucose served as an indicator of cell function with respect to insulin secretion. In the presence of glucose, a significant increase in the cellular calcium levels was evident, thus confirming the rational of the experimental design. The reduction of the intracellular calcium influx by the test substances means a reduction of glucose-stimulated insulin release which was not substantial. Glibenclamide, a sulphonylurea and oral hypoglycaemic drug primarily stimulates insulin secretion by blocking the K-_ATP_ channels and depolarizes the pancreatic β-cells which explains its increased calcium flux^56^. Diazoxide, a benzo thiadiazine derivative operates in contrast to glibenclamide mode of action. The drug exerts its effects through binding to the SUR subunit of the K-_ATP_ channels, resulting in the opening of the channels and increased potassium entry into the cell (hyperpolarisation of the cell membrane) thus inhibiting insulin secretion^57^. It is therefore unexplainable at this time the observed increase in calcium staining by diazoxide. Elevation of the intracellular free calcium concentration is a key event in insulin secretion by pancreatic β-cells. Increased glucose metabolism, a major stimulus to β-cells, ultimately results in elevation of the ATP/ADP ratio causing plasma membrane depolarization through the closing of K-_ATP_ channels. This leads to the entry of calcium through the opening of voltage-gated Ca^2+^ channels, which are located on plasma membranes^58^. Since mitochondria produce the most of glucose-derived ATP, mitochondrial dysfunction will impact negatively on glucose-stimulated insulin secretion. The summary of the results is presented in Table 3.

**Table 3:** Summary of the results.

Assay	Triclosan (T)	4-tert-Octylphenol (O)	Triclosan + 4 tert-Octylphenol (Mix)	Comments
Glucose-galactose differential cytotoxicity	Increased cytotoxicity in galactose medium indicative of potential mitochondrial toxicity	Higher IC_50_ in galactose than in glucose	Increased sensitivity to galactose at lower concentrations but more resistant at higher concentrations	T conforms to a potential mitochondrial toxin
				O cell death appears independent of mitochondrial involvement
				Mix IC_50_ values are different suggestive of extra mitochondrial toxicity
Glucose uptake	Decreased glucose consumption at high concentration, increase at low concentration but not substantial compared with Blank	Consumption pattern as with T. No significant change in glucose consumption	The increased consumption relative to the blank was not substantial	T and O—no meaningful change in glucose consumption suggested cell death mechanism independent of mitochondrial function
				Mix—increased glucose consumption suggested that the combination enhances mitochondrial dysfunction
Oxidative stress	Dose dependent increase in ROS but variation high hampering statistical significance. Decreased glutathione levels corroborated ROS production	Dose dependent increase in ROS but variation high hampering statistical significance. Decrease glutathione levels corroborated ROS production	Increased ROS, but no clear dose dependency	T—significant reduction in GSH levels correlates with sustained oxidative stress
				O, Mix—Increased GSH was in response to oxidative challenge. Response not statistically significant
				All treatments led to oxidative stress which may be expected as all treatments induced cell death
Annexin PI	TCS induced cell death by apoptosis	Cell death by 4-tOP was by necrosis	Mix induced cell death by apoptosis	Probably TCS and 4-tOP have different molecular targets in INS-1 cells
Caspase 3 activation	Increase in caspase 3 activation not substantial	Increase in caspase 3 activation not substantial	Statistically significant increase in caspase 3 activation	Unlike the Mix, apoptotic cell death by TCS was not caspase mediated
Mitochondrial mass	No substantial change in mitochondrial content at all the concentrations relative to the untreated cells	No substantial change in mitochondrial content at all concentrations relative to the untreated cells	No substantial change in mitochondrial content at all concentrations	The substantial increase in the mitochondrial mass was recorded for Mel and Etop in connection with increased ROS activity
Mitochondrial membrane potential MMP	Significant decrease in MMP at the high concentration. only	The increase in MMP was not substantial	Significant decrease in MMP at the high concentration	Loss of MMP suggests potential mitochondrial toxin
				O, cell death independent of mitochondrial function, correlates with necrotic cell death
				Robust decrease in MMP at higher concentration suggests possible synergistic interaction or secondary effect such as apoptosis. Caspase and mitochondrial toxicity in galactose medium seem to support apoptosis rather than a direct mitochondrial effect
Intracellular calcium levels	The reduction in glucose stimulated intracellular calcium flux was not substantial	No significant changes in glucose stimulated intracellular calcium levels	Reduction in glucose stimulated intracellular calcium flux at lower concentration though not substantial	T: Decreased efficacy of glucose stimulated intracellular calcium correlates with mitochondrial dysfunction as observed for MMP and glucose consumption data
				O: No significant effect on glucose stimulated calcium influx, however a significant increase in cellular calcium was evident in the absence of glucose which may influence the reliability of this data
				Mix: Data does not correlate directly with that observed for MMP and glucose consumption. A complex interaction. Diazoxide and Glibenclamide significantly increased intracellular Calcium flux

## Conclusion and future direction

The study appears to support previous report that TCS is a weak mitochondrial toxin^6^ that combined exposure of INS-1 to the two compounds did not result in synergistic but probably antagonistic effects. The results confirmed apoptosis as the mode of cell death via a mitochondria-mediated pathway by TCS and Mix. Contrariwise, 4-tOP induced cell death by necrosis which was not mediated by mitochondrial pathway. Although the two compounds have been reported to be oestrogenic^59,60^ but they seemed to have targeted different sub cellular molecules in INS-1 cell. The adverse impact of the compounds on β-cell function probably provides an insight into the environmental role in the aetiology of non-communicable diseases like diabetes. While TCS acts mitochondrially, the molecular target of 4-tOP in INS-1 cell still needs to be precisely defined. The recorded increase in Ca^2+^ influx by diazoxide which contrasted with its primary role of inhibiting insulin secretion need be re-investigated.

## References

[1] Mimoto MS, Nadal A, Sargis RM (2017). Polluted pathways: Mechanisms of metabolic disruption by endocrine disrupting chemicals. Curr. Envir. Health Rpt..

[2] Nagel SC (1997). Relative binding affinity-serum modified access (RBA-SMA) assay predicts the relative in vivo bioactivity of the xenoestrogens bisphenol A and octylphenol. Environ. Health Perspect..

[3] Heindel JJ (2017). Metabolism disrupting chemicals and metabolic disorders. Reprod. Toxicol..

[4] Li Z, Zhang H, Gibson M, Li J (2012). An evaluation on combination effects of phenolic endocrine disruptors by estrogen receptor binding assay. Toxicol. In Vitro.

[5] Ajao C (2015). Mitochondrial toxicity of triclosan on mammalian cells. Toxicol. Rep..

[6] Weatherly LM (2016). Antimicrobial agent triclosan is a proton ionophore uncoupler of mitochondria in living rat and human mast cells and in primary human keratinocytes. J. Appl. Toxicol..

[7] Tolosa L, Gómez-Lechón MJ, Donato MT (2015). High-content screening technology for studying drug-induced hepatotoxicity in cell models. Arch. Toxicol..

[8] Shariff A, Kangas J, Coelho LP, Quinn S, Murphy RF (2010). Automated image analysis for high-content screening and analysis. J Biomol. Screen.

[9] Yang R, Li N, Ma M, Wang Z (2014). Combined effects of estrogenic chemicals with the same mode of action using an estrogen receptor binding bioassay. Env. Toxicol. Pharmacol..

[10] Xia P (2016). Functional toxicogenomic assessment of triclosan in human HepG2 cells using genome-wide CRISPR-Cas 9 screening. Environ. Sci. Technol..

[11] Mantovani A (2016). Endocrine disrupters and the safety of food chains. Horm. Res. Paediatr..

[12] Beck, B.D., Calabrese, E.J., Slayton, T.M. & Rudel, R. The use of toxicology in the regulatory process. In *Principles and Methods of Toxicology* (ed. Hayes, W A) 89 (InformaHealhcare USA, Inc., 2008).

[13] Loewe S, Muischnek H (1926). Effect of combinations: Mathematical basis of problem. Arch. Exp. Pathol. Pharmakol..

[14] Ribeiro E, Ladeira C, Viegas S (2017). EDCs mixtures: A stealthy hazard for human health?. Toxics.

[15] Orlicka-Płocka M, Gurda-Wozna D, Fedoruk-Wyszomirska A, Wyszko E (2020). Circumventing the Crabtree effect: Forcing oxidative phosphorylation (OXPHOS) via galactose medium increases sensitivity of HepG2 cells to the purine derivative kinetin riboside. Apoptosis.

[16] Rossignol R (2004). Energy substrate modulates mitochondrial structure and oxidative capacity in cancer cells. Cancer Res..

[17] Sanuki Y, Araki T, Nakazono O, Tsuru K (2017). A rapid mitochondrial toxicity assay utilizing rapidly changing cell energy metabolism. J. Toxicol. Sci..

[18] Swiss R, Niles A, Cali JJ, Nadanaciva S, Will Y (2013). Validation of HTS-amenable assay to detect drug—induced mitochondrial toxicity in the absence and presence of cell death. Toxicol. In Vitro.

[19] Bestman JE, Stackley KD, Rahn JJ, Williamson TJ, Chan SSL (2015). The cellular and molecular progression of mitochondrial dysfunction induced by 2,4-dinitrophenol in developing zebrafish embryos. Differentiation.

[20] Shepherd PR, Kahn BB (1999). Glucose transporters and insulin action–implications for insulin resistance and diabetes mellitus. N. Engl. J. Med..

[21] Reyhanoglu, G. & Tadi, P. Etoposide. *Natl Lib Med* https://www.ncbi.nlm.nih.gov/books/NBK557864/ (2022).

[22] Song Z (2015). Curcumin improves high glucose-induced INS-1 cell insulin resistance *via* activation of insulin signalling. Food Funct..

[23] Moon SJ, Dong W, Stephanopoulos GN, Sikes HD (2020). Oxidative pentose phosphate pathway and glucose anaplerosis support maintenance of mitochondrial NADPH pool under mitochondrial oxidative stress. Bioeng. Transl. Med..

[24] Marroquin LD (2007). Circumventing the crabtree effect: Replacing media glucose with galactose increases susceptibility of HepG2 cells to mitochondrial toxicants. Toxicol. Sci..

[25] Garvey WT (1998). Evidence for defects in the trafficking and translocation of GLUT4 glucose transporters in skeletal muscle as a cause of human insulin resistance. J. Clin. Invest..

[26] Pi J (2007). Reactive oxygen species as a signal in glucose-stimulated insulin secretion. Diabetes.

[27] Hassan ZK (2012). Bisphenol A Induces hepatotoxicity through oxidative stress in rat model. Oxid. Med. Cell. Longevity.

[28] Nandi D, Patra RC, Swarup D (2005). Effect of cysteine, methionine, ascorbic acid and thiamine on arsenic-induced oxidative stress and biochemical alterations in rats. Toxicology.

[29] Huang J, Tan PH, Tan BK, Bay BH (2004). GST-pi expression correlates with oxidative stress and apoptosis in breast cancer. Oncol. Rep..

[30] Wang J, Wang H (2017). Oxidative stress in pancreatic beta cell regeneration. Oxid. Med. Cell. Longevity.

[31] Reinehr R, Sommerfeld A, Keitel V, Grether-Beck S, Haüssinger D (2008). Amplification of CD95 activation by caspase 8-induced endosomal acidification in rat hepatocytes. J. Biol. Chem..

[32] Schutte B, Nuydens R, Geerts H, Ramaekers F (1998). Annexin V binding assay as a tool to measure apoptosis in differentiated neuronal cells. J. Neurosci. Methods.

[33] Demchenko AP (2013). Beyond annexin V: Fluorescence response of cellular membranes to apoptosis. Cytotechnology.

[34] Fabisiak JP, Tyurina YY, Tyurin AA, Lazo JS, Kagan VE (1998). Random versus selective membrane phospholipid oxidation in apoptosis: Role of phosphatidylserine. Biochemistry.

[35] Tran DN, Jung EM, Yoo YM, Jeung EB (2020). 4-tert-Octylphenol exposure disrupts brain development and subsequent motor, cognition, social, and behavioral functions. Oxid. Med. Cell. Longevity.

[36] Qian J (2006). Octylphenol induces apoptosis in cultured rat sertoli cells. Toxicol. Lett..

[37] Kim SK, Lee HJ, Yang H, Kim HS, Yoon YD (2004). Prepubertal exposure to 4-tert-octylphenol induces apoptosis of testicular germ cells in adult rat. Arch. Androl..

[38] Kim SK (2006). Nonylphenol and octylphenol induced apoptosis in human embryonic stem cells is related to Fas-Fas ligand pathway. Toxicol. Sci..

[39] Phan T-N, Marquis RE (2006). Triclosan inhibition of membrane enzymes and glycolysis of streptococcus mutans in suspensions and biofilms. Can. J. Microbiol..

[40] Dinwiddie MT, Terry PD, Chen J (2014). Recent evidence regarding triclosan and cancer risk. Int. J. Environ. Res. Public Health.

[41] Martinez MM, Reif RD, Pappas D (2010). Detection of apoptosis: A review of conventional and novel techniques. Anal. Methods.

[42] Kagan VE (2000). Oxidative signaling pathway for externalization of plasma membrane phosphatidylserine during apoptosis. FEBS Lett..

[43] Vermes I, Haanen C, Steffens-Nakken H, Reutelingsperger CA (1995). novel assay for apoptosis: Flow cytometric detection of phosphatidylserine expression on early apoptotic cells using fluorescein labeled Annexine V. J. Immunol. Methods.

[44] Jamil S, Lam I, Majd M, Tsai SH, Duronio V (2015). Etoposide induces cell death via mitochondrial-dependent actions of p53. Cancer Cell Int..

[45] Norberg E, Orrenius S, Zhivotovsky B (2010). Mitochondrial regulation of cell death: Processing of apoptosis-inducing factor (AIF). Biochem. Biophys. Res. Commun..

[46] Shi Y (2004). Caspase activation, inhibition, and reactivation: A mechanistic view. Protein Sci..

[47] Salvesen GS, Duckett CS (2002). IAP proteins: Blocking the road to death’s door. Nat. Rev. Mol. Cell Biol..

[48] Lin Y (2013). Exposure to bisphenol A induces dysfunction of insulin secretion and apoptosis through the damage of mitochondria in rat insulinoma (INS-1) cells. Cell Death Dis..

[49] Presley AD, Fuller KM, Arriaga EA (2003). MitoTracker Green labeling of mitochondrial proteins and their subsequent analysis by capillary electrophoresis with laser-induced fluorescence detection. J. Chromatogr. B. Anal. Technol. Biomed. Life. Sci..

[50] Rostambeigi N (2011). Unique cellular and mitochondrial defects mediate FK506- induced islet β-cell dysfunction. Transplantation.

[51] Yadav N (2015). Oxidative phosphorylation-dependent regulation of cancer cell apoptosis in response to anticancer agents. Cell Death Dis..

[52] De Filippis B (2015). Mitochondrial free radical overproduction due to respiratory chain impairment in the brain of a mouse model of Rett syndrome: Protective effect of CNF1. Free Radical Biol. Med..

[53] Nemade H (2018). Cell death mechanisms of the anti-cancer drug etoposide on human cardiomyocytes isolated from pluripotent stem cells. Arch. Toxicol..

[54] Spies L, Koekemoer TC, Sowemimo AA, Goosen ED, Van de Venter M (2013). Caspase-dependent apoptosis is induced by Artemisia afraJacq. ex Willd in a mitochondria-dependent manner after G2/M arrest. South Afr. J. Botany.

[55] Bauer TM, Murphy E (2020). Role of mitochondrial calcium and the permeability transition pore in regulating cell death. Circ. Res..

[56] Seena PT, Sreejesh PG, Thampi BSH, Sreekumaran E (2017). Hypoglycaemic effect of glibenclamide: A critical study on the basis of creatinine and lipid peroxidation status of Streptozotocin-induced Diabetic rat. Indian J. Pharm. Sci..

[57] George P, McCrimmon R (2012). Diazoxide. Pract. Diabetes.

[58] Gembal M, Gilon P, Henquin JC (1992). Evidence that glucose can control insulin release independently from its action on ATP-sensitive K+ channels in mouse B cells. J. Clin. Invest..

[59] Wang L (2012). Monitoring of selected estrogenic compounds and estrogenic activity in surface water and sediment of the Yellow River in China using combined chemical and biological tools. Environ. Pollut..

[60] Lee HR, Choi KC (2013). 4-tert-Octylphenol stimulates the expression of cathepsins in human breast cancer cells and xenografted breast tumors of a mouse model via an estrogen receptor-mediated signaling pathway. Toxicology.

